# Percutaneous extraction of retained guidewire from a 22-year-old woman 5 years after atrial tachycardia ablation: A novel application of the hangman loop snare technique

**DOI:** 10.1016/j.hrcr.2024.09.006

**Published:** 2024-09-12

**Authors:** Robert M. Tungate, Anil Tiwari, Pranav M. Patel, Theodore Cruz Bryan

**Affiliations:** 1 Division of Cardiology, Department of Medicine, University of California, Irvine Medical Center, Orange, CA; 2 Division of Cardiothoracic Anesthesiology, Department of Anesthesiology and Perioperative Care, University of California, Irvine Medical Center, Orange, CA; 3 Department of Radiological Sciences, University of California, Irvine Medical Center, Orange, CA

**Keywords:** Retained guidewire, Retained object, Guidewire extraction, Atrial tachycardia ablation, Lead extraction, Quality and safety in ablation

**Key Teaching Points**

• The hangman loop snare technique is an established extraction technique that can be applied to the percutaneous extraction of a retained venous guidewire and mechanical lysis of venous endovascular adhesions, even after years of retention.
• Recommended practices to prevent retained foreign bodies after endovascular procedures in the electrophysiology laboratory include routine education and competency testing of all team members, adhering to standardized safety checklists during vascular procedures, requiring 2 people to verify guidewire removal during each procedure, and routinely reviewing post-procedure chest radiography.
• The Joint Commission has designated retained guidewire a sentinel safety event that should be investigated with a root cause analysis.

## Introduction

We present a case of delayed diagnosis and successful endovascular extraction of a retained vena cava J-tip 0.035-in guidewire from a 22-year-old woman 5 years after atrial tachycardia ablation using the hangman loop snare technique.

## Case report

The patient first experienced palpitations when she was 10 years old. By age 16 years, external event monitoring revealed episodes of orthodromic atrioventricular nodal re-entrant tachycardia up to 260 beats per minute. These episodes lasted for up to 30 minutes at a time and were associated with dizziness, shortness of breath, and palpitations. Her symptoms improved, but did not resolve, with metoprolol. An echocardiogram was without significant cardiac structural abnormalities. Electrocardiogram revealed a prolonged QTc interval of 489 milliseconds, which normalized appropriately during an exercise stress test. She demonstrated excellent exercise capacity at that time. Examination of procedural documentation revealed that she underwent an uncomplicated ablation of the slow atrioventricular nodal pathway at age 16 years. Her symptoms recurred after the ablation, and she underwent a second external event monitor which demonstrated episodes of atrial tachycardia. She was trialed on flecainide therapy with little improvement. At age 17 years, she underwent an elective atrial tachycardia ablation. Procedure notes indicate that a coronary sinus catheter, a 5-spined radial mapping catheter, and a radiofrequency ablation catheter were advanced via the right femoral vein. Using isoproterenol, premature atrial contractions from the lateral right atrial wall and a focus of sustained atrial tachycardia near the right atrial appendage were each revealed and ablated. Post-ablation testing revealed no recurrence of arrhythmia. According to documentation, all standard safety protocols were observed during the procedure.

At follow-up with her electrophysiologist 2 months later, her palpitations were improved but that she had developed episodic, dull, irregular chest discomfort. She was offered repeat event monitoring but declined.

The discomfort persisted for years and steadily became associated with radiation to the lower abdomen and groin. She sought consultation with multiple physicians. Documentation denoted that one provider ascribed her symptoms to menstrual discomfort, and she underwent pelvic ultrasonography, which was unremarkable. She was treated with baclofen and ibuprofen with little improvement. At age 22 years, approximately 5 years after her last ablation, she was again evaluated at an urgent care clinic. She underwent chest and abdominal radiography, which was interpreted as abnormal, and she was urgently referred to our institution for an evaluation.

**Figure 1:**
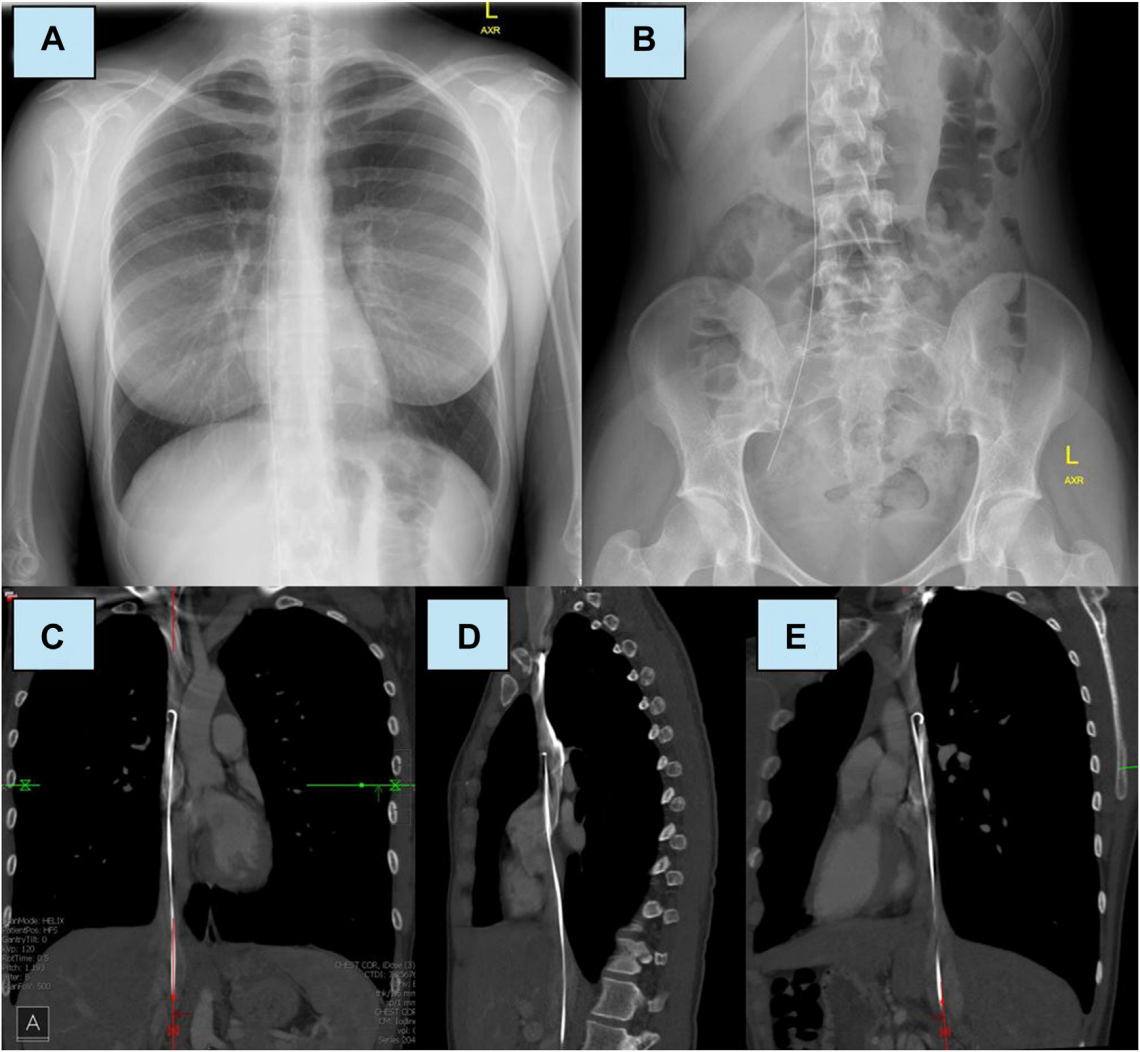
**A:** Chest radiograph on presentation reveals the retained J-wire extending into the superior vena cava along the right edge of the vertebral column. **B:** Abdominal radiograph shows the termination of the retained wire in the femoral vein. **C:** Coronal computed tomography (CT) scan revealing the thoracic course of the retained J-tipped wire. Multiple sections of wire are free-floating within the vein. **D:** Sagittal-plane CT scan showing the same. **E:** Multiplanar reconstruction CT scan showing the same from a posterolateral oblique plane.

Vital signs and physical examination revealed a healthy young woman. Chest and abdominal radiography showed a J-tipped guidewire extending along the right vertebral column from the groin up to the supracardiac thorax (Figure 1). Computed tomography venography demonstrated the course of the wire from the femoral vein to the superior vena cava with adherence of the wire to the wall of the vena cava in some areas (Figure 1).

Her case was reviewed in a multidisciplinary vascular surgery conference. As some areas of the wire appeared free-floating and would be suitable for endovascular extraction, she was scheduled for percutaneous endovascular retrieval under propofol sedation.

**Figure 2:**
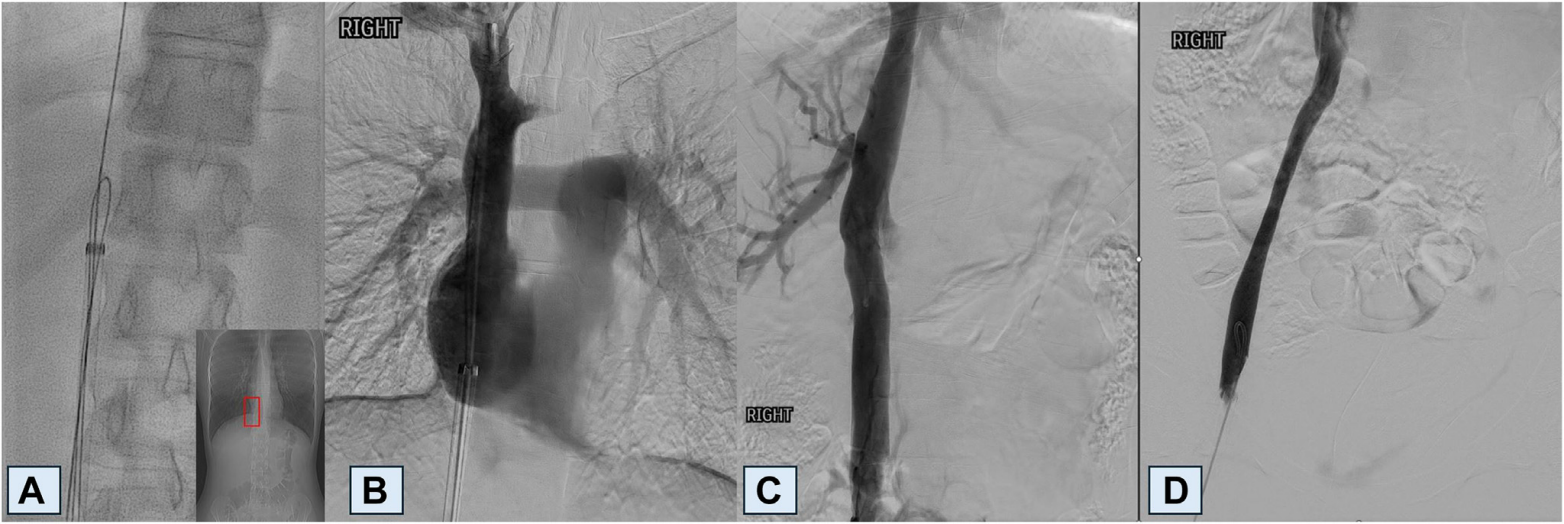
A hydrophilic wire loop (**A**) captured the retained guidewire near the level of the diaphragm. Post-retrieval venogram revealed no injury to the superior vena cava (**B**) or abdominal inferior vena cava (**C**). Narrowing of the right common femoral vein (**D**) was measured as 1.4 cm at the narrowest point. This reflects chronic endothelial thickening provoked by the retained guidewire.

**Figure 3:**
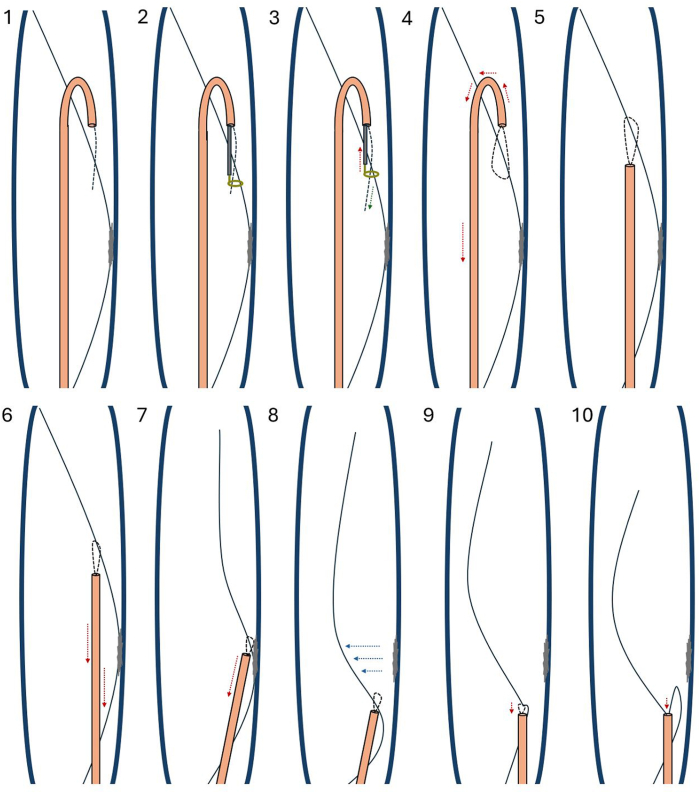
An ideogram depicting the hangman loop snare technique for J-wire extraction in this case. **1:** A hydrophilic wire (*dashed line*) is advanced via a reverse-curve guide catheter (*orange*) to the retained wire (*solid black line*). **2:** An endovascular snare (*gold*) is advanced through the guide catheter and captures the distal end of the hydrophilic wire. **3:** The endovascular snare is withdrawn into the guide catheter. **4:** The reverse-curve guide catheter is withdrawn and straightened. **5:** A hangman loop has captured the object. **6:** The catheter is subtracted along the free segment of wire. **7:** The snare is dragged along the adhered segment to free it from the vascular adhesions (*gray blotch*). **8:** The snare is dragged to the proximal end of the wire, freeing the wire. **9:** The snare is subtracted into the catheter, pulling the wire within. **10:** The wire is removed through the catheter.

In the vascular laboratory, under propofol sedation, a 14F 45-cm sheath was inserted via the right common femoral vein using a modified Seldinger technique. The sheath size was chosen to accommodate forceps if needed. Subsequently, a 5F reverse-curve catheter was advanced into the vena cava via the right femoral vein and venography was performed (Figure 2). The catheter was positioned alongside a free-floating portion of the wire in the central inferior vena cava. A hydrophilic-coated guidewire was advanced through the catheter and looped around the wire. The distal end of the guidewire was captured using an endovascular snare to form a loop snare, reproducing the hangman loop technique previously described for inferior vena cava filter retrieval^1^^,^^2^ (Figure 3). The loop snare was then pulled downward to separate the wire from the wall. Once the snare was near the wire's caudal end, the sheath advanced while the snare was retracted to collapse the 0.035-in wire into the sheath. Completion venogram demonstrated no venous injury. Mild stenosis of the right iliac vein was noted due to endothelization of the guidewire (Figure 2).

The patient was discharged in stable condition on the day of her procedure.

## Discussion

Unintentionally retained guidewire after venous catheterization is, fortunately, an uncommon complication. Nonetheless, as endovascular procedures become more commonplace, the incidence of this preventable complication is rising significantly.^3^ According to a review by the Joint Commission, there were 73 total reported cases between 2012 and 2019, of which 8 were due to cardiac catheterization or interventional radiology procedures,^4^ and we identified at least 1 case of retained wire after a catheter ablation procedure.^5^ Up to 40% of these were not identified until after the patient was discharged from the hospital and 4 patients died from complications due to the retained wire.^4^ The Joint Commission has designated retained wire as a sentinel safety event that requires root cause analysis. Reported causes of this complication include human factors, inadequate or inconsistent adherence to procedural protocols, incomplete intrateam communication, and equipment malfunctions. Nearly one-half of wires migrate within the vasculature,^4^ even as distally as the brain.^5^^,^^6^ We contacted the ablating hospital’s quality department to report the safety event for internal investigation.

Effective, reproducible systems to prevent unintentionally retained guidewire complications in endovascular laboratories are necessary. Patient safety organizations in the United States and United Kingdom recommend routine education and competency testing of team members, a culture of open communication within the procedure team, adhering to standardized checklists during vascular access, requiring 2 people to verify guidewire removal during a procedure, and routine postintervention chest radiography.^4^^,^^7^

Previously published case reports detailed retained guidewires going undiagnosed for up to 2 years.^5^^,^^8^ In this reported case, the retained wire went undiagnosed for approximately 5 years, evading workups conducted by multiple physicians for chest and abdominal pain. Because the wire was retained for so long, there was concern that adhesions between the vasculature and wire may pose an unacceptable risk of vessel injury with percutaneous extraction. Retrospective data from cardiac implantable device lead extraction cases suggested that lead adherence and vascular fibrosis present on preprocedural computed tomography scans predicts increased complexity of an object extraction, necessitating greater fluoroscopy time or more frequent upgrade to specialized snares.^9^ Although multiplanar computed tomography revealed regional wire adherence to the inferior vena cava wall, some segments of the wire were free-floating and thus amenable to fluoroscopy-guided extraction. Multiple techniques for percutaneous extraction with angiography are well-established.^1^ A hangman loop snare technique was selected at a free-floating segment of the wire, as it allowed for the operator to apply tension at a precise wire location (Figure 3) and free the wire from the wall in segments. This contrasts with preformed loop snares, which are better suited to applying tension at the ends of a wire and may cause fragmentation of the wire if it is wall adherent. The technique has been established as effective for IVC filter retrieval, particularly in challenging cases with embedded device segments or with severe filter tilt,^2^ but has not been previously applied to venous guidewire extraction or lysis of vascular adhesions to our knowledge.

Postretrieval angiography revealed an excellent result without major venous trauma or perforation. Narrowing of the femoral vein was attributed to subacute inflammation and fibrosis due to repeated trauma from the wire.

## Conclusion

We presented a case of delayed diagnosis and successful endovascular extraction of a retained vena cava 0.035-in J-tip guidewire from a 22-year-old woman 5 years after atrial tachycardia ablation using the hangman’s loop snare technique.

## Funding Sources

This research did not receive any specific grant from funding agencies in the public, commercial, or not-for-profit sectors.

## Disclosures

The authors have no conflicts of interest to disclose.
